# Memristor-Based Edge Detection for Spike Encoded Pixels

**DOI:** 10.3389/fnins.2019.01386

**Published:** 2020-01-17

**Authors:** Daniel J. Mannion, Adnan Mehonic, Wing H. Ng, Anthony J. Kenyon

**Affiliations:** Department of Electronic and Electrical Engineering, University College London, London, United Kingdom

**Keywords:** memristor, edge detection, computer vision, spiking neural networks, neuromorphic computing

## Abstract

Memristors have many uses in machine learning and neuromorphic hardware. From memory elements in dot product engines to replicating both synapse and neuron wall behaviors, the memristor has proved a versatile component. Here we demonstrate an analog mode of operation observed in our silicon oxide memristors and apply this to the problem of edge detection. We demonstrate how a potential divider exploiting this analog behavior can prove a scalable solution to edge detection. We confirm its behavior experimentally and simulate its performance on a standard testbench. We show good performance comparable to existing memristor based work with a benchmark score of 0.465 on the BSDS500 dataset, while simultaneously maintaining a lower component count.

## 1. Introduction

Interest in the application of memristors was originally driven by their potential as non-volatile memory elements. Subsequent work has demonstrated their capability to compute, to emulate biological synapses, and even to perform some of the functions of the biological neuron. Resistance switches, a sub-class of memristors, can be seen as devices that switch between two or more discrete resistance states. However, they exhibit very rich resistance dynamics under a variety of electrical stimuli. Here we demonstrate that it is not necessary to fully switch such devices to obtain useful functionality. They can be operated in an analog regime to perform elementary computing tasks: in our example, edge detection, and potentially far more. This suggests the possibility of reconfigurable networks of memristors in which different sections of an array can simultaneously store data, perform Boolean logic, generate spikes, integrate multiple inputs, and perform a variety of machine intelligence-related tasks. The memristor thus becomes a simple two terminal building block for a reconfigurable set of computing architectures.

When we consider memristors as computational elements it is largely as accelerators of mathematical operations such as the dot product operator. These accelerations then lead to the speed up of conventional algorithms further down the line. In this work we take the premise a step further by showing that a unique combination of volatile device behaviors with a potential divider arrangement accelerates not just a single operation but the entire computational problem of edge detection in a single stage.

Before detailing previous memristor based studies, we should acknowledge that the field of low power vision is a well researched field with promising alternatives such as event cameras (Gallego et al., 2019). Event cameras encode images in a different manner than a typical charge-coupled device. Rather than reporting the absolute value of a pixel, an event camera signals when the change in a pixel's value exceeds a threshold. Encoding images in this manner when combined with absolute pixel values, allows for more efficient algorithms one example being the edge detection and tracking of Kueng et al. (2016). The technique can be considered a novel sensor technology requiring further processing by a central processing unit (CPU) or graphics processing unit (GPU) to handle the unique data produced by the camera.

In contrast, memristor based techniques work with absolute pixel values and are intended as hardware accelerators with the aim of reducing computational overhead. Alone they will struggle to achieve the same performance as event cameras, which exploit the sophisticated processing possible on a CPU/GPU. For example, in the tracking work of Kueng et al. (2016) edge detection is achieved through a combination of both a Harris corner detector and a Canny edge detector. Therefore, memristor techniques should not be considered the end solution but instead as accelerators to be used in conjunction with other systems.

The work presented in this paper follows the latter approach in that it works with absolute pixel values and is intended as a hardware accelerator. However, it differentiates itself from existing techniques in both function and form. Firstly, it does not simply accelerate a single operation, such as a crossbar does for the dot product operation, but instead accelerates the entire process of edge detection. The output requires no additional processing except for the reading of spikes. Secondly, we replace the commonly used crossbar structure, which is the standard in memristive image processing, and instead use a potential divider built from our volatile devices. The combination of volatile devices placed in a potential divider arrangement is unique and has not been used before in the application of image processing. It differs entirely from studies using non-volatile devices in a crossbar (Yakopcic and Taha, 2017; Khokhar and Khalid, 2018; Li et al., 2018), differing in both device behavior and circuit layout. Where potential dividers have been used before in image processing, they have been non-volatile and required the frequent reprogramming of weights. For example, the study that most closely resembles our own approach is the use of memristive threshold logic to detect moving objects (Maan et al., 2015). Although their memristors are also in a potential divider arrangement their use is more complicated. They are non-volatile devices requiring a training phase between frames, in which their conductances are reprogrammed depending on the previous frames values. In contrast, our approach requires no programming nor training phase, instead operating on the fly. This makes for a simpler circuit design.

We will begin by outlining the origin and basis of the existing memristor based techniques and then detail how our approach differs.

In conventional computing there exist a number of algorithms to carry out edge detection, one example being the Sobel algorithm (Duda, 1973). In this the gradient across neighboring pixels is calculated from the scalar dot product of the pixel in question and its surrounding pixels with a predetermined 3 × 3 matrix, referred to as a kernel. Two different kernels are used, one determining the horizontal gradient, *G*_*x*_ and the other the vertical gradient, *G*_*y*_.

$$\begin{array}{ll} G_{x}=\left[\begin{array}{ccc} -1 & 0 & +1\\ -2 & 0 & +2\\ -1 & 0 & +1 \end{array}\right] & G_{y}=\left[\begin{array}{ccc} +1 & +2 & +1\\ 0 & 0 & 0\\ -1 & -2 & -1 \end{array}\right] \end{array}$$

The results of these two dot products are combined to find the overall gradient using Equation (1).

(1) $$\begin{array}{l} G=\sqrt{{G_{x}}^{2}+{G_{y}}^{2}} \end{array}$$

As a result, Sobel relies on a number of dot product operations being carried out across the image. However, because dot products involve the passing of data back and forth between processors and memory they lead to bottlenecks and inefficiencies (Fatahalian et al., 2004). Therefore, one approach to improving the efficiency of edge detection is to accelerate the dot product operator.

Circuits designed to accelerate dot product operations are named dot product engines (DPEs). An effective DPE can be implemented using the memristor (Chua, 1971; Strukov et al., 2008). The memristor is a two terminal device similar to a resistor with the exception that its conductance is not fixed. Instead it can be adjusted with an applied voltage. For example, voltages in one polarity may make the device more conductive while voltages in the opposite polarity will make it less conductive. Therefore, a memristor's conductance depends on the potentials applied to it in the past and so can be considered a form of memory.

This memory property is exploited when implementing memristive DPEs. Memristors are arranged into a crossbar array with the value of one matrix element encoded in the conductance of the memristor and the value of the second encoded in the applied voltage (Alibart et al., 2013). The multiplication of matrix elements is carried out by applying the voltage to the memristor producing an output current as defined by Ohm's law. As is required in the dot product operator, the output currents for each element are summed in accordance with Kirchoff's current law. Crossbar arrays consisting of memristors have previously been shown to be effective DPEs (Hu et al., 2016).

The Sobel algorithm was implemented by Can Li et. al. on a memristive DPE with good performance (Li et al., 2018). They made use of non-volatile, analog memristors in a crossbar structure. A variation on directly implementing Sobel on a DPE is to instead teach the same algorithm to a neural network. Memristive crossbar arrays are still used as DPEs. However, the multiplication is of inputs and trained network weights, not of the matrices directly derived from Sobel. Yakopcic et al. constructed a multilayer perceptron network and trained it to replicate the Sobel algorithm, again with good performance (Yakopcic and Taha, 2017). Their system used non-volatile memristors with 128 discrete conductance states. More bespoke implementations depart further from the conventional crossbar or neural network architectures and replace the dot product entirely. Fuzzy XOR gates implemented with memristors can determine pixel gradients (Merrikh-Bayat et al., 2014) and swarm computations, based on the behavior of ants, have been replicated with grids of memristors (Pajouhi and Roy, 2018).

Although these approaches aim to improve efficiency through changes to circuit design, none consider the encoding of their signals, instead choosing to use only continuous real encoding. An alternative is to use spike-encoded signals. Information is represented in either spike timings, spike shape, or both. A spike-based circuit is typically analog and computes at the arrival of a spike. It is argued that its inactivity between spikes can result in a more power efficient network (Joubert et al., 2012). This has led to the development of neural networks that exploit such spiking signals, called spiking neural networks (SNNs), with a variety of CMOS implementations (Indiveri et al., 2006). Spiking neural networks operate using unsupervised learning rules, one example being the spike-timing-dependent plasticity rule (STDP). Applications such as character recognition via STDP have been demonstrated in both Von-Neumann computer systems (Diehl and Cook, 2015) and memristive systems (Covi et al., 2016). However, computation is not solely restricted to the use of STDP learning rules. Memristors exhibiting generic analog behavior have been used in the learning of spatiotemporal patterns and sound localization (Wang et al., 2018) as well as in the sorting of spike patterns (Werner et al., 2016).

In our application, pixel values are encoded into the frequency of spike trains. Therefore, the detection of edges is equivalent to determining the difference in frequency between neighboring pixels. We present a simple potential divider circuit consisting of two amorphous silicon oxide memristors whose switching characteristics we have detailed previously (Mehonic et al., 2017; Munde et al., 2017; Kenyon et al., 2019). The potential divider design is able to indicate the difference in frequency of its inputs with the amplitude of spikes at its output. By inserting our memristive potential divider between neighboring pixels we can detect differences in pixel values and in turn identify edges.

We will begin by describing the devices used in this work and how their behavior differs from typical memristors. We then go on to detail our circuit and experimental data confirming its behavior. Finally, we describe our model of the circuit and present simulated results for a collection of images.

## 2. Devices

**Figure 1:**
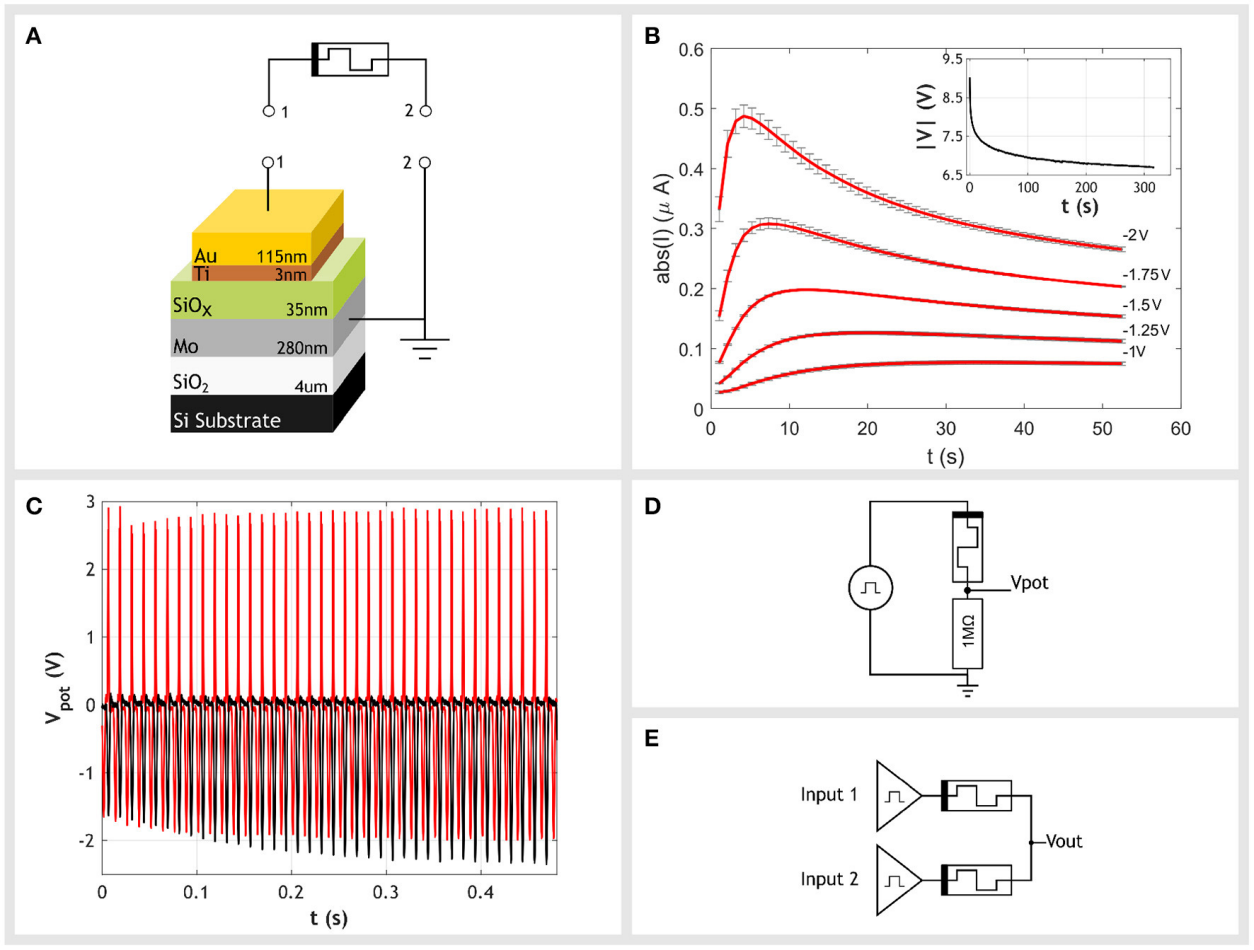
**(A)** Our devices use an active layer of 35 nm sputtered amorphous silicon oxide. The bottom contact is a 280 nm layer of molybdenum and the top contact is a 115 nm layer of gold with a 3 nm wetting layer of titanium. **(B)** Examples of the current transients which occur when constant negative voltages are applied to the top electrode with respect to the bottom electrode. Transients consist of two parts. There is an initial increase in conductance and a subsequent decrease. In this work we operate only within the first region, the increase in conductance. Inset is a plot of the absolute voltage across the device during the initial stressing stage. A constant current of −10μA is driven through the device. The applied voltage decreases over time, indicating the reduction in device resistance that occurs as a result. **(C)** Negative and positive voltages have an opposite effect on the device's conductance. When a train of negative voltage spikes were applied to the device in a potential divider setup with a fixed 1*MΩ* resistor, the voltage of spikes at the output increases over time (black trace), corresponding to an increase in conductance of the memristor. In contrast, when a train of positive spikes are interleaved in anti-phase with the negative spikes (red trace), the output voltage increases to a lesser extent. This demonstrates the competing effects positive voltages have on the memristor. The positive spikes are reversing the changes in conductance cause by the negative spikes. **(D)** Setup to demonstrate the competing effects of negative and positive polarities. Gaussian pulses with a full width at half maximum (FWHM) of 1.3 ms are generated by a signal generator. These are applied to the top contact of the memristor which is in a potential divider with a fixed 1*MΩ* resistor. The output voltage, *V*_pot_, is measured at the output of the potential divider. **(E)** Our circuit that determines the difference in frequency of two input spike trains. Both inputs generate pulse trains with a negative amplitude and a frequency proportional to their input value. Each input is connected to a single memristor. Both memristors then join at a common node. The output of the circuit, *V*_out_, is taken at this common node. The amplitude of output spikes indicate the difference between the two input frequencies. Larger differences in frequency result in larger amplitudes at the output.

Our devices are of a metal-insulator-metal (MIM) structure with a sputtered silicon oxide insulator layer. They consist of a gold top electrode with a wetting layer of titanium on the oxide and a bottom electrode of molybdenum. The device size is 200 × 200 μ*m*. Figure 1A details the dimensions of each layer. More details regarding fabrication can be found in Mehonic et al. (2017).

These devices were originally developed as binary memory cells, able to switch between two distinct low and high resistance states (Mehonic et al., 2017) but have also demonstrated a number of neuromorphic uses such as in replicating synapse functionality (Zarudnyi et al., 2018), neuronal spiking and integration (Mehonic and Kenyon, 2016) as well as more conventional machine learning techniques such as interference (Mehonic et al., 2019). A typical application is as elements within random access memories, referred to as resistive random access memory (RRAM). RRAM devices switch between their two distinct resistance states in response to a sufficiently large voltage being applied to the device. Although we have previously shown our devices behave in this manner we do not use this conventional RRAM behavior in this work. Instead, we use an analog operating mode which is obtained through a change in the initial stressing of the device.

### 2.1. Analog Operation and Current Transients

It is well known the behavior of a memristor is defined by its device history. One of the key stages in this history is the initial stressing of the device, in which the device transitions from a pristine to an operational state. This initial stressing is typically carried out using a voltage sweep and is referred to as electroforming. After electroforming, the device exhibits binary switching behavior as we have shown in Mehonic et al. (2017). However, by modifying this initial stressing we find the device can be forced into an alternative operating mode, one that does not exhibit discrete jumps in resistance but instead analog and volatile changes. A characteristic feature of this operating mode is the observed transient in current in response to constant potentials, shown in Figure 1B. Therefore, we have devices able to exhibit either digital or analog behaviors depending on how the device is initially stressed.

In order to induce the analog operating mode the device is not electroformed with a voltage sweep but instead has a constant current driven through the device at the top electrode. The magnitude of the stressing current can range from -10 to −100μA and should be maintained until the change in device conductance slows and levels out. An example of this forming process is included as an inset to Figure 1B. It should be stressed this is not an operating condition but an initial step in order to induce the analog regime and so could be considered a kind of electroforming. However, it should not be confused with the electroforming typically associated with memristors. This process is a smooth transition, very different from the discrete jumps of electroforming. After removing the current bias and allowing the device to relax, it is now in the analog regime and will exhibit the characteristic current transients.

Transients similar to those in Figure 1B have been documented before in other MIM structures. Studies of barium strontium titanate capacitors (Zafar et al., 1998; Saha and Krupanidhi, 2001) revealed transients of the same form and timescale. Although it may seem reasonable to believe transients are the result of capacitive charging this theory fails in two ways. Firstly, the change in conductance is in the opposite sense to what would be expected. If we were simply charging the electrode we would expect a decrease in current, instead we observe an initial increase. Secondly, the latter half of the transient, which is a decrease in current, occurs over a duration of 50s. Considering our device is driven by a low impedance voltage source, we would expect significantly shorter timescales. The capacitance of the device can be approximated to be 35 to 40 pF; assuming a relative dielectric constant between 3.5 to 4, an area of 4 × 10^−4^(cm^2^) and a thickness of 35 nm. If we then assume the combined source and lead resistances were to be at the extreme end, say 100 Ω, the time constant of the system would range from 3.5 to 4 ns, many orders of magnitude smaller than what is observed. Instead, the cause of this behavior is thought to be drifting oxygen vacancies in turn modulating electronic conduction (Meyer et al., 2005; Zhong et al., 2010).

When driven with a negative bias at the top electrode, current transients consist of two parts; an initial increase in conductance followed by a later decrease. The increase in conductance is volatile and resets on the order of seconds, whereas the decrease in conductance is more persistent. In this work, we operate within only the first portion of the transient. Within this region, applying a negative bias to the top electrode creates an increase in the device conductance which can then be reversed with subsequent positive biases. This behavior is demonstrated in Figure 1C. A single device was placed in series with a fixed 1 MΩ resistor connected to ground, forming a potential divider - as shown in Figure 1D. Any change in device conductance is observed in the change of the potential divider's output voltage. An increase in device conductance will result in larger output amplitudes while a decrease in conductance will result in smaller output amplitudes. When a train of pulses with negative amplitudes is applied to the device (black trace) we observe an increase in the amplitude of output spikes over time, corresponding to an increase in device conductance. In contrast, when a positive pulse train is interleaved in anti-phase with the original negative train (red trace) the two processes begin to compete. The negative pulses increase conductivity while the positive pulses decrease it. Although this still leads to a small increase in conductivity, which we assume is due to some asymmetries, it is less than when the negative train does not face competition. It is this competing behavior between spike trains of opposite polarities that forms the basis of our circuit.

## 3. Circuit Design

To determine the gradient across two neighboring pixels we require a circuit that detects the difference in frequency between two spike trains. Our circuit achieves this by exploiting the opposing effects spike trains of opposite polarities have on our devices.

Both input sources are connected to each other through a combination of two memristors in series, as shown in Figure 1E. The memristors are in opposite orientations with their bottom contacts connected, forming a potential divider. The amplitudes of output spikes are therefore dependent on the conductances of both memristors. Sources produce spikes of negative polarity and are connected to the top contact of their respective memristor. For either memristor, when the source directly connected to it generates a spike and the neighboring source is grounded, it experiences a negative bias, causing an increase in conductance. In direct contrast, the second memristor, whose source is grounded, is in the opposite orientation and so experiences a bias in the opposite polarity. This causes its conductance to decrease. We have therefore introduced a form of competition between the two inputs. When a source fires it acts to increase the conductance of its attached memristor while decreasing the conductance of its neighbor.

**Figure 2:**
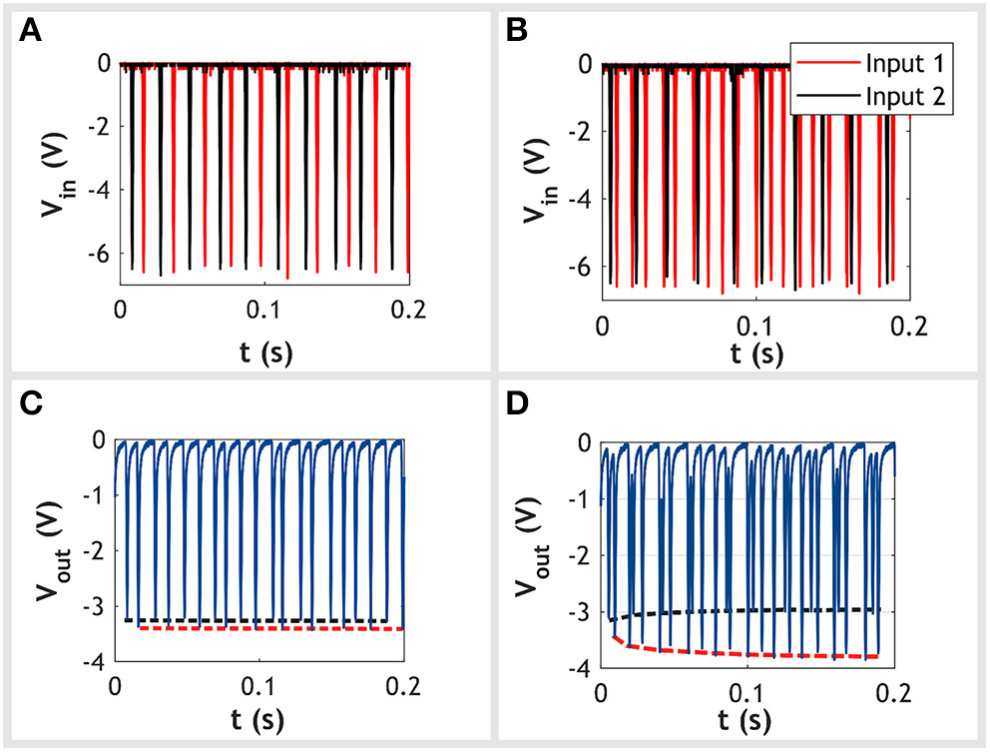
Experimental data demonstrating the circuit's ability to detect differences in frequency between two input spike trains. Two scenarios are presented: the first with no difference in input frequency and the second with a difference of 50 Hz. Spikes are Gaussian shaped with a full width at half maximum (FWHM) of 1.3 ms. Each Gaussian pulse has been cropped to a width 2 ms. **(A,C)** These show the input and output signals, respectively, for the case of no difference in input frequency. Both input 1 (red trace) and input 2 (black trace) are set to 50 Hz. In the plot beneath we see the amplitude of spikes at the output remain approximately constant for both inputs. For clarity we have included an envelope tracking the output spikes caused by input 1 (dotted red trace) and input 2 (dotted black trace). **(B,D)** Shows the input and output signals, respectively, for the case with a 50 Hz difference in input frequency between the two inputs. Input 1 is set to 100 Hz while input 2 remains at 50 Hz. For clarity, we have again overlaid two envelopes tracking the output spikes caused by input 1 and input 2. This time, we observe at the output that spikes caused by input 1 increase in amplitude over time, whereas those from input 2 remain constant.

If the two inputs are of the same frequency any increase in conductance caused by one input is swiftly canceled out by the opposing effect of the other. This will result in both memristors having a similar conductance with no change in output amplitude. Alternatively, when one input has a higher frequency than the other, the high frequency input will overpower the opposing effect of the second input. This will drive the memristor with the high frequency input to become more conductive while suppressing increases in the conductance of the low frequency input. Given the potential divider arrangement, the amplitude of output spikes for the high frequency input will increase while those of the low frequency spikes are driven down to a minimum value. This behavior is shown in Figure 2. The inputs are initially both set to 50 Hz with no observed change in the amplitude of output spikes. The frequency of one input is then set to 100 Hz, resulting in a difference in frequency of 50 Hz. This causes the amplitude of the output spikes caused by the 100 Hz input to increase in amplitude, whereas the output spikes generated by the 50 Hz signal remain at their initial value. Therefore, as the difference in frequency between the two inputs increases, so does the amplitude of output spikes from the higher frequency input. These amplitudes can be used as an indicator for the difference in input frequencies.

Importantly, at no point should the two inputs be allowed to fire at the same time. If this occurs the circuit would no longer behave as a potential divider due to neither of the inputs being grounded. As a result the output voltage will merely follow the voltage of both inputs producing an erroneous output. In this work we avoid conflicts by inhibiting the latter spike when two spikes do happen to overlap. We chose this approach because it was considered the simplest to implement in a physical system. Each spike source would be designed with an enable/disable input, which, when triggered, inhibits any output. The output of a source would then connect to its neighbor's enable/disable input. Thus, when a source fires and produces a spike, it is simultaneously inhibiting its neighbor from firing. Crucially, this implementation uses only local signals, avoiding issues with the routing of control signals.

**Figure 3:**
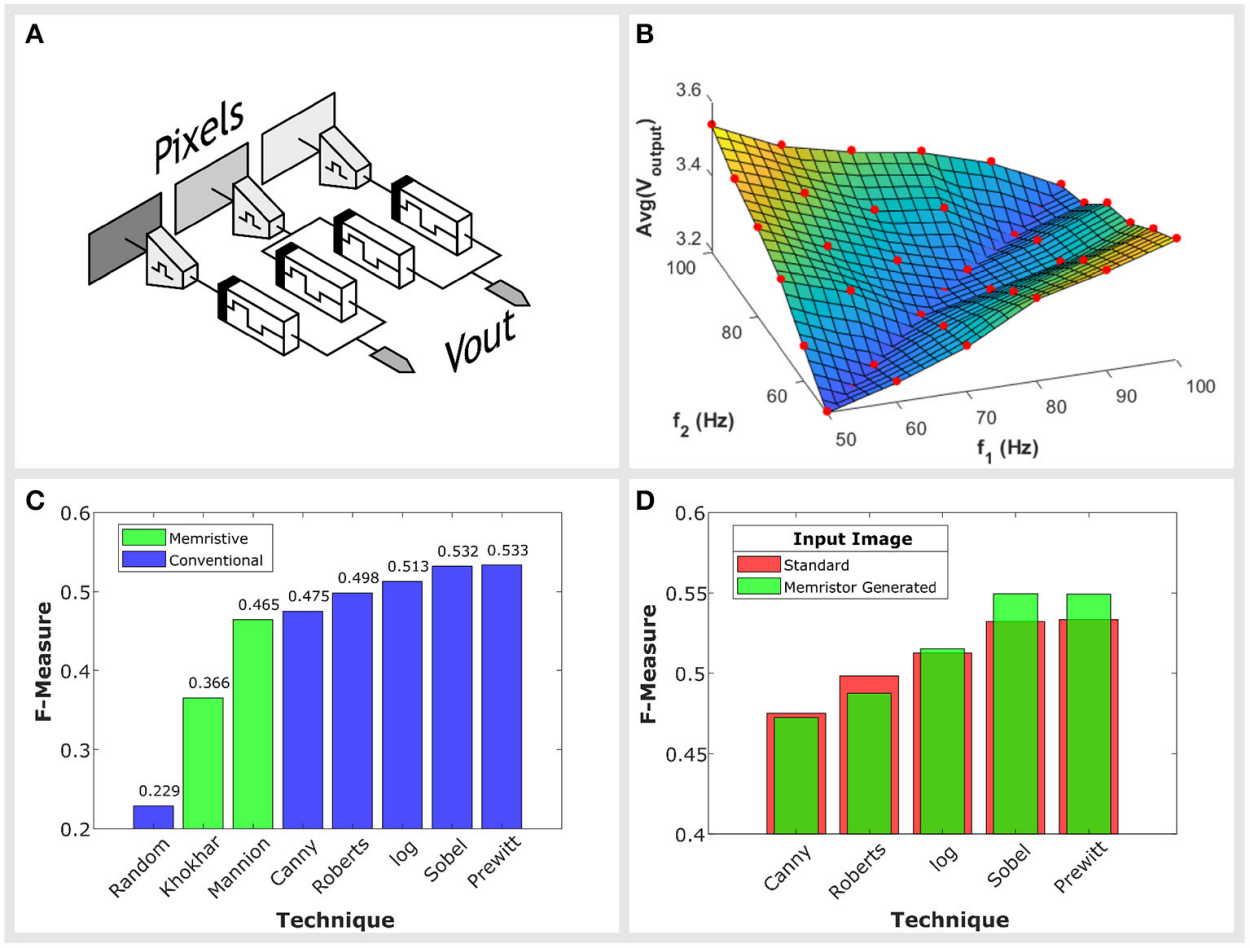
**(A)** An illustration of how edge detection would be implemented. The circuit is placed between two neighboring pixels. Large differences in pixel values will produce output spikes with larger amplitudes. **(B)** The look-up map describing our circuit's behavior. The average amplitude of output spikes above a threshold is plotted along the *z* axis. We use this look-up table during simulations. It approximates the circuit's output for any given pair of input frequencies. The sampling points from which this map was interpolated from are illustrated with red circles. **(C)** Benchmarking results on the BSDS500 dataset. The distribution of F-Measures, defined in Arbeláez et al. (2011) are plotted for memristive techniques (green) and standard operators (blue). The results are obtained from the set of 200 test images provided by BSDS500. **(D)** Comparison of F-Measure scores for a set of operators using both the original test images and images produced by our own circuit as their input. An improvement in performance is observed over the Prewitt, Sobel, and log operators.

Edges manifest as sharp changes in pixel values across the image, equivalent to large differences between the frequencies of neighboring pixels. By connecting our circuit between two neighboring pixels, as shown in Figure 3A, we detect these differences and produce output spikes with amplitudes proportional to the differences in frequency. Large amplitude output spikes correspond to sharp changes in pixel values, indicating potential edges.

## 4. Methodology

### 4.1. Simulation

In order to simulate the circuit's performance when applied to an image, we require a model approximating its behavior. We constructed a look-up table describing the circuit's response. Given two input frequencies, the look-up table returns the average amplitude of spikes at the circuit's output. These measurements were taken after the circuit was allowed to settle, always ≤ 500 ms after inputs were first applied. Spikes take the same form as those used in Figure 2, they are Gaussian in shape with a full width at half maximum (FWHM) of 1.5 ms and are trimmed to a width of 2 ms. The model was constructed using data obtained from a physical implementation of the circuit with input frequencies ranging from 50 to 100 Hz. We characterize the circuit with a sampling resolution of 10 Hz. Each of the points sampled are illustrated with a red circle in Figure 3B and are used to form the dataset for interpolation. The look-up table produced as a result of this process is shown in Figure 3B.

**Figure 4:**
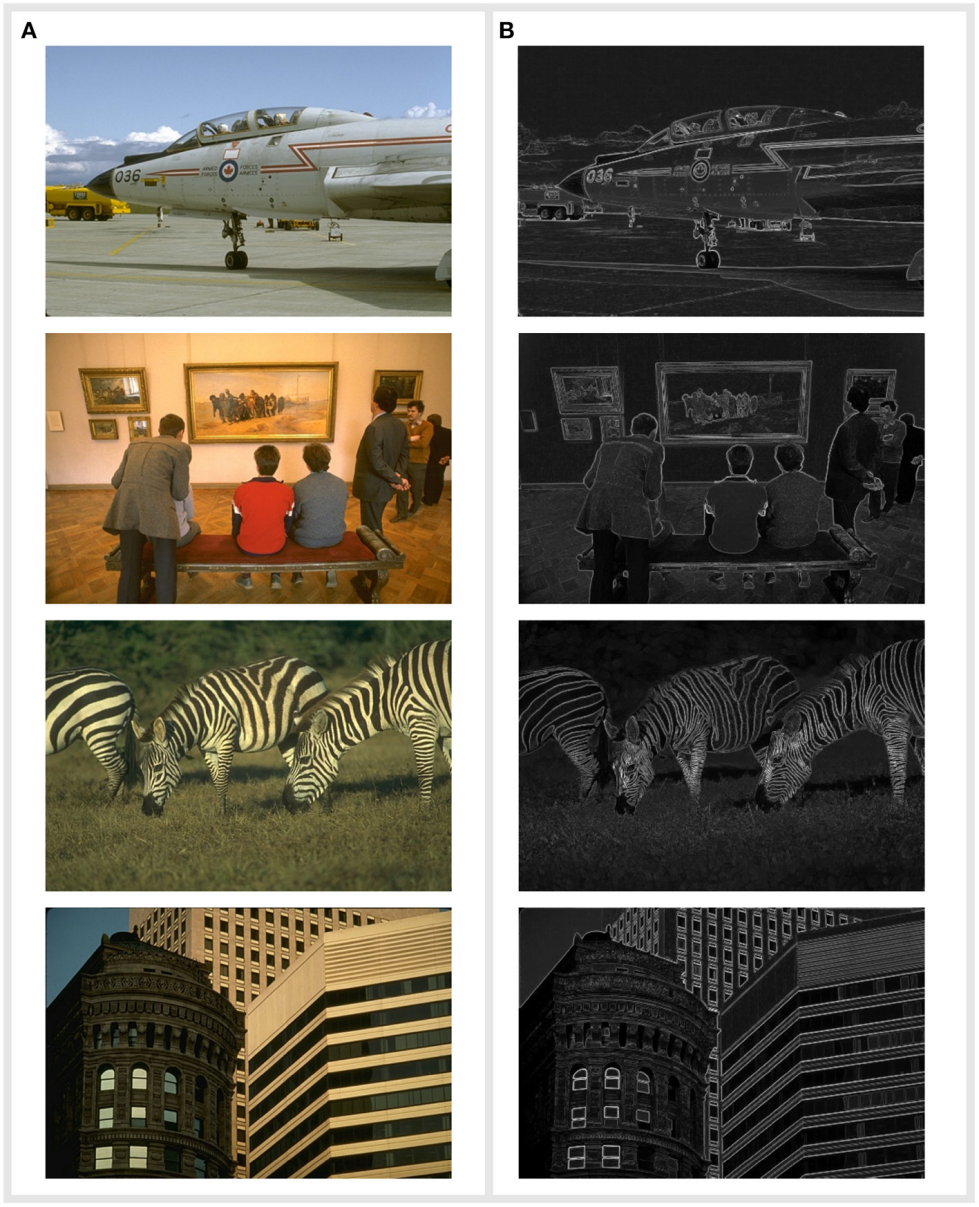
**(A)** A sample of the original input images presented to the circuit. Source: Arbeláez et al. (2011). In simulation, there exists an edge detection circuit between each neighboring pixel. Pixels are mapped from their 0–255 value to a frequency range between 50–100 Hz. **(B)** The corresponding output images of the simulation. Each pixel represents the output of an edge detection circuit placed between two neighboring pixels. The average amplitude of output spikes above a threshold is mapped from the voltage to a pixel value from 0 to 255 and is plotted in this image. Brighter pixels indicate edges. We have combined the simulations of edge detection in both the vertical and horizontal plane.

Input frequencies are generated from images such as those presented in Figure 4A. Pixel values are first converted from color to grayscale using MATLAB's rgb2gray function. The function uses the following equation to combine the three components: 0.2989*R*+0.5870*G*+0.1140*B* where *R, G*, and *B* correspond to the red, green and blue components of the pixel. The grayscale values are then linearly mapped from 0 to 255 to frequencies between 50 and 100 Hz. Example outputs for each simulation are shown in Figure 4B. In this figure we have combined the results from both a horizontal and vertical edge detection. Each pixel represents a single circuit placed between two neighboring pixels. The value of the pixel is proportional to the average amplitude of output spikes that are above a defined threshold - the same quantity as that returned from the look-up table. Brighter pixels correspond to larger output amplitudes, which are caused by larger differences in input frequencies and therefore indicate potential edges.

### 4.2. Benchmarking

Benchmarking is a useful tool in comparing solutions to a computational problem. Of the previous memristive edge detection studies, only one makes use of benchmarking (Khokhar and Khalid, 2018), with the BSDS500 dataset (Arbeláez et al., 2011). The BSDS dataset provides 500 images for testing edge/boundary detection algorithms combined with a benchmarking script to standardize comparisons between algorithms. Although the authors make use of the dataset, they do not use the associated benchmarking script. Instead using their own custom analysis. We make use of both the dataset as well as its benchmarking script in the hope that future studies can compare effectively against our work. The authors of Khokhar and Khalid (2018) have kindly made their data available for us to put through the standard benchmark as a comparison.

The performance of each memristor implementation is compared against a number of standard operators: Prewitt, Sobel, log, Roberts and Canny. Each technique is awarded a score, named the F-Measure. This score is related to the probability of a pixel being an edge and the probability of a false positive with more details on its derivation given in Arbeláez et al. (2011). The larger the F-Measure the more effective the edge detection. In Figure 3C we have plotted the F-Measure scores for each technique, in addition to a random approach which merely classifies pixels as edges with a 50% probability.

In addition to this, we also characterize the use of our circuit as an input to standard edge detection operators, quantifying whether or not it improves performance. For each operator, we begin by running the benchmarking script using the original dataset images as inputs to form a set of control data. We then run a second test but instead now use the output image generated by our circuit as the input image to the conventional operator. The performances of these two cases are then compared to identify any improvements in performance.

## 5. Results and Discussion

### 5.1. Performance

Examples of the circuit's output are presented in Figure 4B. Unfortunately, it is not possible to quantitatively compare our circuit against the techniques of Li et al. (2018) and Yakopcic and Taha (2017). Both studies use different images and in the case of Li et al. (2018), their input has purposefully been made to exhibit noise. However, in using the BSDS500 dataset we are able to compare the circuit's performance against other conventional operators as well the memristor based work of Khokhar and Khalid (2018) who also make use of this dataset.

Figure 3C shows our circuit's benchmark performance against other techniques, where we achieve an F-Measure of 0.465. This places our performance at the bottom end of conventional operators, on par with the Canny operator. However, in comparison to other memristive techniques, such as Khokhar and Khalid (2018) who achieve an F-measure of 0.366, we present a jump in performance.

An alternative approach would to consider our circuit an accelerator, for example, as the input to one of the standard operators. This technique leads to an improvement in performance for the Prewitt, Sobel and log operators as shown in Figure 3D. On the other hand, algorithms that score lower F-Measures on the BSDS500 dataset, such as the Roberts and Canny detectors, do not improve through the use of our circuit as an input.

### 5.2. Variance

Variability in device performance is a common issue with memristive devices. In this work, we are primarily concerned with the variance in device resistances leading to offsets in voltage at the potential divider's output and in turn define a maximum tolerable variability.

Our device resistances varied from 0.77 to 2.17 *M*Ω when sampled across 16 devices, with 50% of devices falling within the resistance range of 0.99–1.66 *M*Ω. We found these variations were spatially distributed, neighboring devices would have similar resistances while those separated were likely to vary.

**Figure 5:**
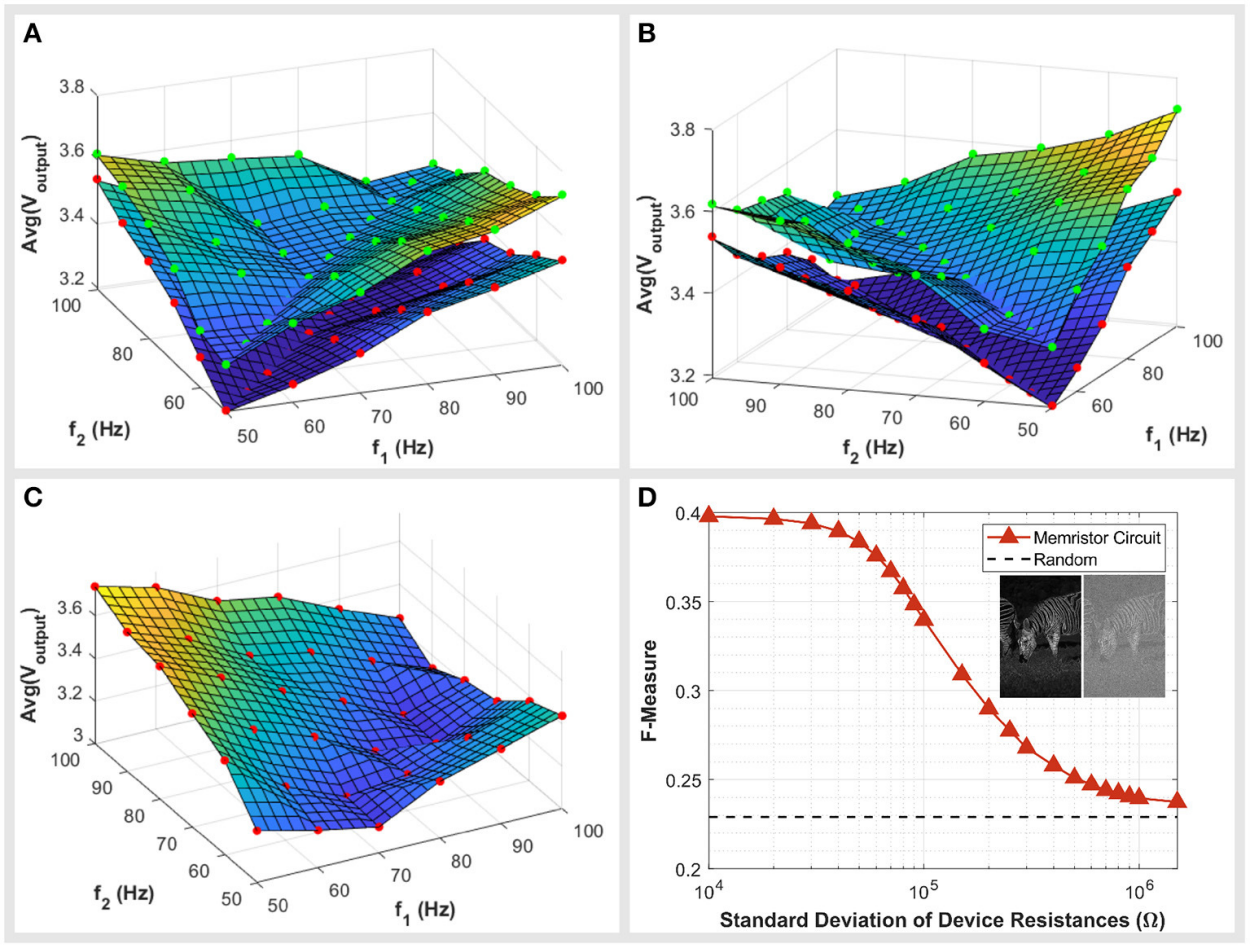
**(A,B)** The circuit response plotted in two different perspectives for clarity. The response for two circuits are presented in both figures, a potential divider of two high resistance devices (red markers) and a potential divider of two low resistance devices (green markers). Markers represent the inputs sampled for the circuit. **(C)** The circuit response when devices have asymmetric resistances. The red markers indicate the points sampled from the circuit. **(D)** The effect of device variance on circuit performance. The circuit's benchmark score (F-Measure) is plotted for a range of simulations where variations in device resistances were introduced. Device variations were distributed randomly and with a Gaussian distribution. The standard deviation of device resistances were increased with the effect of a reduction in performance. For reference the score of a system randomly categorizing pixels as edges is also plotted.

We consider two scenarios when assessing the effect of variance on circuit performance. The first is when both devices of the potential divider have a similar resistance. The second is when the two devices have different resistances. When the two devices making up the potential divider have similar resistances, we find little differences in the circuit behavior other than an offset in spike amplitudes. Figure 5A shows the circuit's response for two instances, a pair of 1.24 and 1.32 *M*Ω devices and pair of 2.17 and 2.10 *M*Ω devices. Figure 5B shows the same plot from a different perspective for clarity. The shape of the circuit's response does not vary significantly between each case, whereas there is a noticeable offset. Alternatively, when pairs of devices are not equal and instead have asymmetric resistances the circuit has an asymmetric response, as shown in Figure 5C. Fortunately, the asymmetry of the circuit's response does not have a significant impact on the circuit performance, with benchmark scores dropping from an F-Measure of 0.474 to 0.459 when we account for such effects.

On the other hand, symmetric variances affect the circuit in a more significant way. The voltage offsets caused by such variances interfere with the classification of edges. This is because a pixel is deemed an edge if the output voltage is above a threshold, however, voltage offsets cause a blurring of this threshold. Some non-edges are raised above the threshold and some edge pixels are dragged beneath the threshold, introducing errors in the output image. Such offsets can be caused by additive thermal noise, investigated in the Supplementary Material, or by device variances which we will now detail.

To quantify the effect of variances on the circuit performance, we simulate the circuit with varying device resistances. Devices exhibit a gaussian distribution of resistances allocated randomly across the image. These variances lead to voltage offsets at the outputs of each circuit, an example of which is included as an inset of Figure 5D. We simulated and benchmarked the system for a range of distributions with varying standard deviations. This resulted in a drop of performance as shown in Figure 5D. The system performs no better than the random control beyond approximately a standard deviation of 250 kΩ. Through this we can define a maximum acceptable standard deviation by using the score of other memristive studies as a threshold. Taking Khokhar and Khalid (2018) as the threshold with a score of 0.366, we can determine a maximum allowable standard deviation of 50 kΩ. The standard deviation of our current devices can be approximated to be 472 kΩ, although this should be treated with caution seeing as we have only 16 samples to characterize hence the approach is not statistically significant. At this stage, it appears the variance in our devices is too large for the system to be realized. Although we cannot comment on the specific cause of such variances, if this were the result of sample fabrication, then it is a matter of refining fabrication processes. However, a more detailed study would have to be carried out to identify the causes of such variances and the ultimate limitations.

### 5.3. Scalability

Our chosen figure of merit to compare the scalability of techniques considers the number of components required for each additional raster/pixel added to the circuit. This includes both the number of additional memristors as well as any periphery circuitry included at output or intermediate layers. This quantifier allows for a quick comparison regardless of whether a scanned or parallel approach is taken. We do not consider the input circuitry. Table 1 compiles the component count per raster for each of the studies cited in this paper. Although data could be collected for most techniques it was not possible to fully assess (Khokhar and Khalid, 2018). We approximate their output to require a single comparator per raster in order to threshold outputs as stated in their paper. However, they also require peripheral circuitry to regularly update memristor weights, which they do not document. This will act to increase both the component count and circuit footprint, hence, we can say with some confidence it is a more complex circuit than the others presented here.

**Table 1:** Comparison of the increase in component count required for each additional implemented raster.

**Study**	**Memristors per raster/Pixel**	**Operational amplifiers per raster/Pixel**	**Size of raster**
Mannion et al. (this study)	+2	+1 (Comparator)	1 × 2
Li et al. (2018)	+25	+1 (Transconductance)	5 × 5
Yakopcic and Taha (2017)	+461	+40 (20 Summing, 20 Unity Gain)	3 × 3
Khokhar and Khalid (2018)	+225	Not available	1 × 2

Of the remaining two techniques by Li et al. (2018) and Yakopcic and Taha (2017), the approach of Li et al. is by far superior with respect to component count. This is not surprising considering their approach features a single crossbar array with one transimpedance amplifier per column whereas Yakopcic's neural network consists of 10 input neurons, 20 hidden layer neurons and 2 memristors for each synapse connection to represent +/- weights. Equally, Yakopcic's hidden neurons consist of two operational amplifiers, further increasing circuit size.

When comparing our circuit to these three techniques we must consider how the system will be implemented. Either a scanned approach may be taken, whereby only a single kernel is physically implemented and then scanned across the image, or a parallel approach is taken, with multiple copies of the circuit operating in parallel, each on their respective section of the image. The scanned approach favors scenarios where latency is less of a concern and small footprints are desired, whereas the parallel approach suits scenarios requiring the real time processing of images. Our circuit requires approximately a twelfth of the memristors required by Can Li yet the same number of output operational amplifiers, albeit in a different configuration. That said, our circuit should not be considered the better technique solely on this basis. As detailed in the following section on limitations, our circuit has a finite settling time due to the memory properties of our devices. Therefore, if a scanning implementation is being used we require a finite relaxation time between the presentation of inputs to avoid mixing. As a result, a scanning approach would favor a crossbar technique such as Li et al. (2018) whereas our circuit is better suited to a parallel implementation.

### 5.4. Limitations

**Figure 6:**
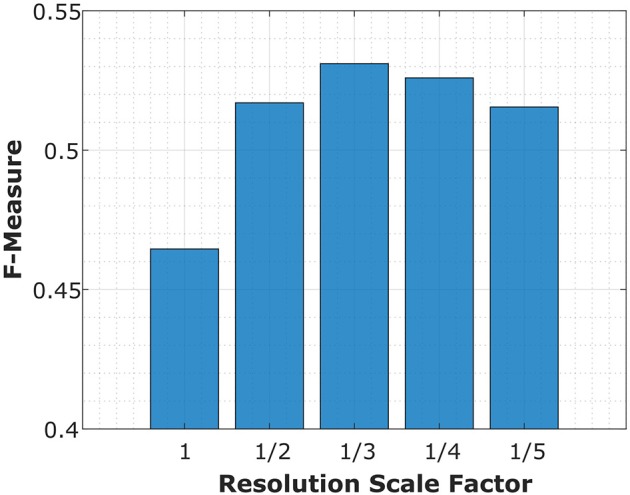
The effect of image resolution on circuit performance. The benchmarking score (F-Measure) is plotted against the scale factor of the image resolution. An improvement in performance is observed for lower resolution images. For example, the highest score occurred when the images were at a 1/3 of their original resolution. It was not possible to investigate the effect of increasing image resolution as there were no high resolution images available for the dataset.

Although our circuit provides the advantage of a potentially reduced component count, we identify some limitations. The first applies to any technique determining the gradient across neighboring pixels and it concerns the resolution of the image. For high resolution images, a sharp edge may occur across a number of pixels. The change in intensity associated with the edge is now spread across the group of pixels, thereby reducing the change experienced by each individual pair of pixels, essentially smoothing out the edge. The higher the resolution, the worse this effect will be. A simple solution is to down-sample the image. However, although this will help in some cases, the image resolution will always play a role in limiting which edges can be detected. We investigate this limitation by reducing the resolution of the benchmark images, processing the image and then scaling the image back to its original resolution for benchmarking. The scores of the circuit for different resolutions are plotted in Figure 6. When the image is reduced in resolution and then applied to the circuit the performance is generally better than at the benchmark's original resolution. However, if the resolution is reduced beyond a third of its original, the performance drops owing to the loss in information that is occurring.

The second limitation is specific to our approach. Once inputs are applied to the circuit, the output has a finite settling time after which it is then stable. The output should not be read before this time to avoid incorrect readings. This limits the operating frequency of the circuit. The configuration used in this work has a settling time consistently ≤ 500 ms. This time can be adjusted through changes in a number of parameters including the amplitudes or widths of input spikes and the chosen operating frequencies.

## 6. Conclusion

We have shown how a potential divider of two memristors can indicate the difference in frequencies between two spike trains. We confirmed this behavior experimentally and applied the circuit to the problem of edge detection successfully achieving a jump in performance compared to other memristive techniques. The circuit requires no external control signals, training signals or power supply, instead operating exclusively on input signals. This proves an advantage for scalability. Without the need for these external signals, as required with DPEs or neural networks, we have reduced the complexity of routing signal paths and computational overhead. Equally, its passive nature combined with spike operation makes it well suited for low power applications. Besides edge detection, the circuit may also have broader applications. Its fundamental behavior is the detection of differences in frequency between two input spike trains. This may prove useful in other computational schemes.

Furthermore, in showing an alternative operating region devoid of switching has computational uses, we have demonstrated yet another function resistance switching devices can provide. With the very same devices able to implement arrays of memory and both analog and discrete computations, we envisage reconfigurable networks of these devices having real potential in delivering flexible hardware accelerators.

## Data Availability Statement

The datasets generated for this study can be found in UCL's data repository: doi: 10.5522/04/9741722.

## Author Contributions

DM carried out the experimental work, built simulations and prepared the manuscript. AM devised experiments and prepared the manuscript. WN fabricated the devices used in the characterization of our circuit. AK supervised the project and prepared the manuscript.

### Conflict of Interest

The authors declare that the research was conducted in the absence of any commercial or financial relationships that could be construed as a potential conflict of interest.
