# Supplementary material for: Memristor-Based Edge Detection for Spike Encoded Pixels
Source: Front Neurosci. 2020 Jan 17;13:1386. doi: 10.3389/fnins.2019.01386 (PMC6978841; doi:10.3389/fnins.2019.01386)
Supplement: Supplementary file 1 [file Data_Sheet_1.PDF]

frontiers

Supplementary Material

1 EFFECT OF ADDITIVE THERMAL NOISE

Whether a pixel is deemed an edge or not depends on whether the corresponding output voltage is above a voltage threshold. For that reason, additive thermal noise risks blurring this boundary and corrupting pixels on the edge of this threshold. We characterise how the circuit's performance degrades with increasing additive thermal noise.

Additive thermal noise originating at the signal generators will adjust the voltage at the output of the potential divider. When an input generates a spike, the output voltage of the potential divider is given by equation S1, where V_1 corresponds to the amplitude of the voltage spike generated by the spiking input. Although the device resistances vary during operation, it is only by a small amount, and so we approximate them as constant throughout the simulation. This allows us to simplify the ratio $R_2/\left(R_1+R_2\right)$ to equal 0.5, assuming devices are of similar value[1]. The additive noise of the spiking input, v_{t1}, and of the grounded input, v_{t2}, are introduced in series with their respective signal generator, resulting in equation S2. From equation S2, we see that the original clean output voltage, $V_{\text{out}} = 0.5 \times V_1$, is corrupted additively by $V_{\text{noise}} = 0.5 \times [v_{t1} - v_{t2}] + v_{t2}$. The additional noise terms, v_{t1} and v_{t2}, are generated randomly and added to each pixel in accordance with equation S2. The system is then benchmarked for a range of random voltage fluctuations.

$$V_{\text{out}} = V_1 \times \frac{R_2}{R_1 + R_2} \tag{S1}$$

$$\begin{aligned} V_{\text{out}} &= 0.5 \times [V_1 + v_{t1} - v_{t2}] + v_{t2} \\ V_{\text{out}} &= V_{\text{clean}} + V_{\text{noise}} \end{aligned} \tag{S2}$$

$$\begin{aligned} \textit{Where}\quad V_{\text{clean}} &= 0.5 \times V_1 \\ V_{\text{noise}} &= 0.5 \times [v_{t1} - v_{t2}] + v_{t2} \end{aligned}$$

In figure S1 we plot the circuit's benchmark score against the magnitude of the random noise at the signal generators. As expected there is a decrease in performance. For smaller magnitudes of noise the system is resilient, with only a small drop in performance. However, beyond a magnitude of 20mV the performance begins to degrade rapidly, eventually approaching the performance of a random system. As a result, we are able to identify the requirements for the system to be realisable. Input voltage sources should exhibit no more than 20mV of randomly fluctuating noise, however, for optimum performance, noise should remain below 10mV.

[1] The effect of varying device resistances has been addressed in the original manuscript.

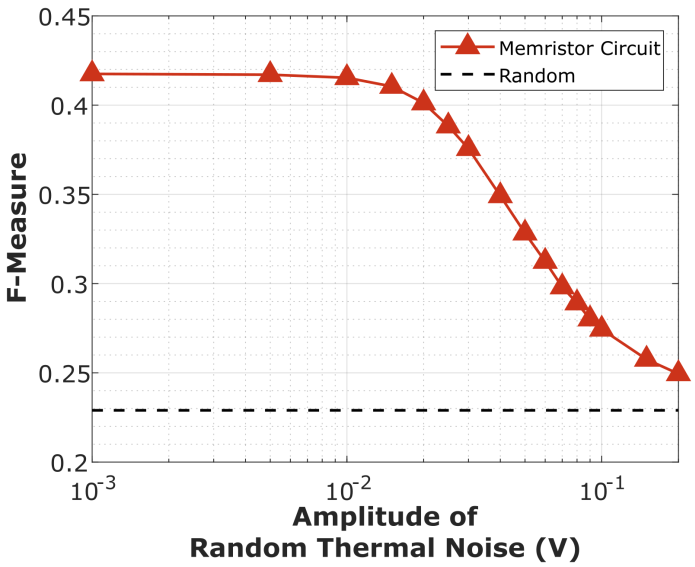

Figure S1. The effect of additive thermal noise on circuit performance. The benchmark score (F-Measure) is plotted against the magnitude of random noise generated at the circuit inputs. The circuit's performance is plotted alongside the performance of a system randomly classifying pixels as edges. As the magnitude of noise increases, the circuit's performance approaches this limit.
